# O.6.2-4 Please stop claiming that elite sport influences the general population to practice physical activity!

**DOI:** 10.1093/eurpub/ckad133.274

**Published:** 2023-09-11

**Authors:** Alexis Lion, Anne Vuillemin, Florian Léon, Charles Delagardelle, Aurélie van Hoye

**Affiliations:** Fédération Luxembourgeoise des Associations de Sport de Santé, L-1445 Strassen, Luxembourg; Association Luxembourgeoise des Groupes Sportifs pour Cardiaques, L-1445 Strassen, Luxembourg; Luxembourg Institute of Research in Orthopedics, Sports Medicine and Science, L-1460 Luxembourg, Luxembourg; Université Côte d’Azur, LAMHESS, F-06200 Nice, France; Foundation for Studies and Research on International Development (Ferdi), F-63000 Clermont-Ferrand, France; Fédération Luxembourgeoise des Associations de Sport de Santé, L-1445 Strassen, Luxembourg; Association Luxembourgeoise des Groupes Sportifs pour Cardiaques, L-1445 Strassen, Luxembourg; Luxembourg Institute of Research in Orthopedics, Sports Medicine and Science, L-1460 Luxembourg, Luxembourg; Department of Cardiology, Centre Hospitalier du Luxembourg, L-1210 Luxembourg, Luxembourg; APEMAC, University of Lorraine, F-54600 Villers-lès-Nancy, France; Department of Physical Education and Sport Sciences, University of Limerick, V94 T9PX Limerick, Ireland; alexis.lion@flass.lu

## Abstract

**Purpose:**

The Paris 2024 Olympic Games Organisation Committee claims that “another impact of the Games on society is reflected in the increase in the practice of sports”. Decision-makers and policymakers still use the trickle-down effect to legitimize spending public money to support elite sport events and/or to finance elite sport programs/athletes. We therefore wanted to investigate if this effect was not a myth.

**Methods:**

We conducted structured Boolean searches across five electronic databases (Pubmed, JSTOR, Web of Sciences, Sportdiscus, and PsycInfo) from January 2000 to August 2021 using the following equation: ((“elite” OR “high level” OR “performance”) OR (“Olympic*” AND *Games”) AND (“sport” OR “athletes” OR “players”)) AND ((“physical activit*” OR “sport” OR “exercise”) AND (“participation” OR “practice”)). We also conducted manual searches using the reference lists of the recovered records. Peer-reviewed studies in English were included if the effects of hosting elite sport events, elite sport success, and elite sport role-modelling on physical activity (PA) or sport practice in the general population were measured.

**Results:**

We identified 12,563 articles and included 36 articles. Most studies used data from the United Kingdom (n = 10), Australia (n = 5), Canada (n = 4), and multiple countries (studies using data from several countries) (n = 4). Seven articles investigated more than one effect of elite sport. Most studies investigated the effect of hosting elite sport events (n = 27), followed by elite sport success (n = 16) and elite sport role-modelling (n = 3). Most studies did not observe an effect of hosting elite sport events, elite sport success, or elite sport role-modelling on PA/sport practice in the general population. We also did not observe any evidence of elite sport effects according to the age range, the geographical scale, or time.

**Conclusions:**

There is no evidence supporting an effect of elite sport in increasing PA or sport participation in the general population. Decision-makers and policymakers should not use the tickle-down effect of elite sport to legitimize spending public money to support elite sport events and/or to finance elite sport programs/athletes. They should invest in other strategies such as those recommended by the World Health Organization.

